# Integrated Cardiopulmonary Profile as an Additive Signal in Patients Evaluated for Suspected Newly Detected Atrial Fibrillation

**DOI:** 10.3390/medsci14040507

**Published:** 2026-08-21

**Authors:** Nilima Rajpal Kundnani, Abhinav Sharma, Ciprian Ilie Rosca, Milan Daniel Velimirovici, Ariana Violeta Nicoras, Dana Emilia Velimirovici, Dragos Cozma, Doina Georgescu

**Affiliations:** 1 Doctoral School, “Victor Babeș” University of Medicine and Pharmacy, Eftimie Murgu Sq. no. 2, 300041 Timișoara, Romania; knilima@umft.ro (N.R.K.); sharma.abhinav@umft.ro (A.S.); milan.velimirovici@umft.ro (M.D.V.); 2 Department of Cardiology, “Victor Babes” University of Medicine and Pharmacy, 300041 Timisoara, Romania; dragos.cozma@umft.ro; 3 Research Centre of Timisoara, Institute of Cardiovascular Diseases, “Victor Babes” University of Medicine and Pharmacy, 300041 Timisoara, Romania; 4 Department V, Internal Medicine I—Discipline of Medical Semiology I, Center of Advanced Research in Cardiology and Hemostasology, “Victor Babeș” University of Medicine and Pharmacy, Eftimie Murgu Sq. no. 2, 300041 Timișoara, Romania; doina.georgescu@umft.ro; 5 Department 1—Nursing, Faculty of Nursing, “Victor Babeș” University of Medicine and Pharmacy, Eftimie Murgu Sq. no. 2, 300041 Timișoara, Romania; 6 General Medicine Faculty, “Victor Babeș” University of Medicine and Pharmacy, Eftimie Murgu Sq. no. 2, 300041 Timișoara, Romania; ariana.nicoras@umft.ro

**Keywords:** atrial fibrillation, NT-proBNP, spirometry, left atrial remodeling, echocardiography, cardiopulmonary profile

## Abstract

Background: Atrial fibrillation (AF) is associated with atrial remodelling, hemodynamic stress, and complex cardiopulmonary interactions. While natriuretic peptides and echocardiographic abnormalities are established markers of AF-related substrate, the contribution of spirometry changes, as a landmark of pulmonary impairment, remains less clearly defined. This study evaluated whether AF is associated with an integrated cardiopulmonary profile characterized by altered spirometry, increased NT-proBNP, and conventional echocardiographic markers of left atrial structure and function. Methods: We performed a retrospective, single-center observational study based on an existing clinical database of adult patients referred for 24 h 12-lead Holter ECG monitoring because of palpitations and/or chest pain. None of the included patients had a previously documented diagnosis of atrial fibrillation before Holter monitoring. Because a single 24 h Holter recording cannot establish spontaneous termination or long-term temporal pattern with certainty, AF documented for the first time during this recording is referred to throughout as “newly detected AF” rather than “paroxysmal AF”. During Holter monitoring, no ECG changes suggestive of myocardial ischemia were detected. From 536 records of patients without previously known AF who underwent 24 h 12-lead Holter ECG monitoring for palpitations and/or chest pain, 164 patients were retained for the main comparison, including 48 with newly detected paroxysmal AF and 116 without AF. Demographic, biochemical, spirometric, and echocardiographic variables were analyzed. The main respiratory parameters were forced vital capacity (FVC), forced expiratory volume in 1 s (FEV1), and peak expiratory flow (PEF). NT-proBNP was the primary biomarker, while conventional left atrial structural and functional variables were used for echocardiographic assessment. Results: NT-proBNP was markedly higher in the AF group (684.5 vs. 62.3 pg/mL, *p* = 0.001), even when accounting for confounders like age, CKD, or CPD, showing by far the largest between-group difference among the biomarkers assessed (formal discrimination statistics such as ROC/AUC were not computed). The exploratory inflammatory markers assessed (hs-CRP, neutrophil/lymphocyte ratio, homocysteine) showed only modest, non-significant differences. Conventional echocardiographic parameters did not differ significantly between groups. In the strict complete-case comparison, spirometric indices did not reach statistical significance; however, preliminary project-level analysis showed lower FVC, FEV1, and PEF in AF patients, but without supporting the COPD diagnosis, and exploratory correlations suggested a relationship between spirometric performance and left atrial function. Conclusions: In this cohort, AF was most strongly associated with NT-proBNP, while the respiratory signal was weaker but directionally consistent with a broader cardiopulmonary phenotype. These findings do not support spirometry or NT-proBNP as stand-alone diagnostic tools for AF. However, when interpreted alongside traditional AF risk factors and symptoms, elevated NT-proBNP with concordant spirometric impairment may represent a hypothesis-generating additive signal supporting longer or repeated rhythm monitoring beyond 24 h in selected patients with suspected AF; this additive value was not formally tested against clinical models alone and requires prospective confirmation.

## 1. Introduction

Atrial fibrillation (AF) is the most common sustained arrhythmia in adults and remains strongly associated with stroke, heart failure, cardiovascular death, and reduced quality of life. Its prevalence continues to rise with population ageing, which makes earlier recognition clinically important, especially before overt complications occur [1,2].

Beyond rhythm disturbance alone, AF reflects a broader process of atrial remodeling, pressure overload, and neurohormonal activation. Among circulating biomarkers, NT-proBNP has shown the most consistent association with prevalent, incident, and screening-detected AF. In cohort studies, natriuretic peptides improved AF prediction beyond standard clinical variables, whereas inflammatory markers generally added less incremental value [3,4,5].

The echocardiographic substrate of AF is equally relevant, but conventional chamber dimensions do not fully describe early atrial disease. Recent imaging studies have shown that left atrial strain predicts incident AF independently of standard morphologic parameters and may remain informative even in patients with normal-sized left atria. This supports the concept that functional atrial impairment can precede overt enlargement and may be more sensitive than routine structural measurements alone [1,6,7].

At the same time, growing evidence suggests that AF is also linked to the respiratory domain. In large population cohorts, lower FEV1 and FVC, as well as both obstructive and restrictive ventilatory patterns, have been associated with higher AF risk after multivariable adjustment. These findings suggest that reduced lung function may reflect a broader cardiopulmonary phenotype rather than isolated pulmonary comorbidity [8,9].

Taken together, these data support a more integrated view of AF. The literature already supports each domain separately, but the combination of cardiac stress biomarkers, atrial remodelling, and spirometric impairment in the same clinical model remains less well characterized; this is an inference from the separate strands of evidence above. In addition, AF patients often display complex comorbidity profiles, which favor multimodal phenotyping over single-marker assessment alone [1,4,9,10,11]. This diagnostic logic is consistent with broader biomedical evidence showing that integrated models may improve clinical interpretation when imaging, functional testing, and biomarkers are considered together rather than separately [12,13].

Therefore, the aim of the present study was to evaluate whether patients with newly detected paroxysmal AF during 24 h 12-lead Holter ECG monitoring exhibit an integrated cardiopulmonary profile characterized by higher NT-proBNP, altered spirometric performance, and conventional echocardiographic markers of left atrial structure and function. Importantly, the study focused on patients without a previous diagnosis of AF who were referred for Holter monitoring because of palpitations and/or chest pain.

## 2. Materials and Methods

### 2.1. Study Design and Setting

This study was designed as a retrospective, observational, single-center analysis based on patients from a tertiary center in western Romania. The aim was to compare patients not previously known to have AF, but for whom it was discovered through symptom-guided ECG-Holter, and those without atrial fibrillation (AF), from the same clinic and comparable background. By comparison, we aim to assess whether AF was associated with a combined profile of spirometric impairment, increased NT-proBNP, and echocardiographic evidence of left atrial remodelling. AF was defined according to the available Holter ECG classification and interpreted in line with contemporary guideline concepts.

### 2.2. Study Population

The source worksheet contained 536 records. Patients were screened according to Holter ECG classification, and only cases with a clear rhythm label suitable for binary comparison were retained for the main analysis. The final study population was divided into two groups: paroxysmal AF and non-AF. Patients with isolated atrial flutter or predominantly ventricular rhythm disturbances were excluded from the primary comparison in order to preserve a cleaner AF-focused model. All patients underwent 24 h Holter ECG monitoring as part of routine ambulatory or inpatient cardiology evaluation for palpitations and/or chest pain; granular hospitalization status and admission indications were not uniformly captured in the source database and are acknowledged as a limitation (Section 4.6).

### 2.3. Holter ECG Monitoring and Rhythm Classification

All included patients underwent 24 h 12-lead Holter ECG monitoring. Holter monitoring was recommended as part of the clinical evaluation of patients presenting with palpitations and/or chest pain. Patients with a previously documented diagnosis of atrial fibrillation were not included in the primary comparative analysis. Therefore, AF cases in the present study represent newly detected paroxysmal AF identified during Holter monitoring. The non-AF group included patients in whom no AF episode was documented during the available Holter recording. During the monitoring period, no ECG changes suggestive of myocardial ischemia were detected.

### 2.4. Inclusion and Exclusion Criteria

Adult patients with available Holter ECG classification and at least one relevant dataset from the respiratory, biochemical, or echocardiographic domain were eligible for inclusion. Records were included if they allowed classification into either the AF or non-AF group and contained usable values for at least one of the variables relevant to the study objectives. All included patients underwent 24 h ECG-Holter due to suggestive symptomatology. No patients had a diagnosis of AF prior to the evaluation.

Exclusion criteria were: missing or non-interpretable Holter classification, duplicate records, isolated atrial flutter, predominantly ventricular rhythm disturbances (frequent ventricular ectopy or non-sustained/sustained ventricular tachyarrhythmia on Holter monitoring), and clearly unusable spirometric or echocardiographic measurements. Because data completeness varied across domains, each statistical analysis was performed on the maximum available number of valid observations for the variables included in that specific comparison.

### 2.5. Objectives and Study Endpoint

The primary endpoint was newly detected atrial fibrillation (AF) documented during 24 h 12-lead Holter ECG monitoring in patients without a previous AF diagnosis. AF episodes were identified as runs of irregular atrial activity without discernible P waves on the Holter tracing. Because a single 24 h recording cannot establish spontaneous termination, minimum episode duration, or long-term temporal pattern with certainty, these cases cannot be formally classified as paroxysmal AF; the term “newly detected AF” (or “first-diagnosed AF”) is therefore used throughout rather than “paroxysmal AF”. For the same reason, absence of AF during a single 24 h recording in the non-AF group cannot exclude intermittent, low-burden AF that was not captured within the recording window, and AF burden and mean ventricular response rate during episodes were not systematically quantified in the source database—both are acknowledged as limitations (Section 4.6).

The secondary objectives were to assess the relationship between spirometric parameters and NT-proBNP, to explore the association between spirometric parameters and echocardiographic indices of left atrial remodeling, and to identify which parameters were most strongly associated with AF within the available retrospective dataset.

### 2.6. Data Collection and Study Variables

The database was reviewed for demographic, biochemical, spirometric, and echocardiographic parameters. Age and sex were collected as baseline variables. The main biochemical variable was NT-proBNP, while hs-CRP, homocysteine, and the neutrophil/lymphocyte ratio were retained as exploratory inflammatory or associated biomarkers.

The respiratory domain focused on FVC (% predicted), FEV1 (% predicted), and PEF (% predicted). These parameters were selected because they were the most consistently available spirometric variables and had already shown a directional association with AF in the preliminary project analysis. The echocardiographic domain focused on conventional markers of left atrial structure and function, including anteroposterior left atrial diameter, left atrial area, left atrial end-diastolic volume, left atrial end-systolic volume, left atrial ejection fraction, and medial e′, where available. Left ventricular systolic and diastolic function parameters were not systematically available in the source database and were therefore not included in the present comparison; this is acknowledged as a limitation, since preserved left atrial size together with markedly elevated NT-proBNP could partly reflect left ventricular diastolic dysfunction rather than an isolated atrial process.

### 2.7. Statistical Analysis

Data entry and cleaning were performed using Microsoft Excel 2016 (Microsoft Corp., Redmond, WA, USA) [14]. Statistical analyses were conducted using IBM SPSS Statistics version 26 (IBM Corp., Armonk, NY, USA) [15] and R version 4.3.3 (R Foundation for Statistical Computing, Vienna, Austria) [16].

Continuous variables were assessed for distributional assumptions and are presented as mean ± standard deviation when approximately symmetric; non-normally distributed variables are presented as median and interquartile range. Categorical variables are presented as counts and percentages. Between-group comparisons (AF vs. non-AF) were performed using Welch’s t-test for continuous variables with approximately symmetric distribution and Mann–Whitney U test for non-normally distributed variables. Categorical variables were compared using the chi-square test, while Fisher’s exact test was used when expected cell counts were small. Correlations between respiratory, biochemical, and echocardiographic variables were assessed using Spearman rank analysis. A two-sided *p* value < 0.05 was considered statistically significant.

A multivariable logistic regression model was used to assess the adjusted association between NT-proBNP and newly detected paroxysmal AF. NT-proBNP was entered after natural-logarithm (ln) transformation because of its skewed distribution; the reported odds ratio corresponds to each 1-unit increase in ln-transformed NT-proBNP [ln(pg/mL)]. The model was adjusted for age, renal dysfunction, and chronic pulmonary disease. Heart failure status, body mass index, and smoking history were not consistently available across the source database and could not be entered as additional covariates; given the modest number of patients with complete data for this model, covariate selection was deliberately kept parsimonious and results should be regarded as exploratory. Adjusted odds ratios, 95% confidence intervals, and *p* values were reported.

### 2.8. Ethical Considerations

The study was conducted in accordance with the Declaration of Helsinki [17] and applicable data protection requirements. Given the retrospective, non-interventional design and the use of anonymized routinely collected clinical data, the requirement for individual informed consent was waived according to institutional policy. The protocol was approved by the Ethics Committee of the Victor Babeș University of Medicine and Pharmacy (approval number: 25, date of approval: 2 March 2026). Data handling complied with the General Data Protection Regulation (EU 2016/679) [18].

## 3. Results

### 3.1. Study Population and Data Availability

All patients retained for the primary comparison had been referred for 24 h 12-lead Holter ECG monitoring because of palpitations and/or chest pain. No patient in the AF group had a previously documented diagnosis of AF before the index Holter recording. Therefore, all AF cases in the present analysis were considered newly detected paroxysmal AF. No ischemic ECG changes were detected during Holter monitoring.

The main worksheet contained 536 patient records. A numeric Holter classification was available for 272 cases, while the remaining records had either data-missing or non-interpretable Holter entries. For the present binary analysis focused on atrial fibrillation, only patients with a clear classification of AF or non-AF on ECG-Holter were retained. This yielded a final comparative cohort of 164 patients, including 48 patients with AF (FiA or FiA+FlA) and 116 patients without AF. Cases with isolated atrial flutter (n = 61) and ventricular events (n = 46) were excluded from the main AF versus non-AF comparison. One additional case with an unclassifiable rhythm category was not suitable for the binary comparison and was excluded.

**Figure 1:**
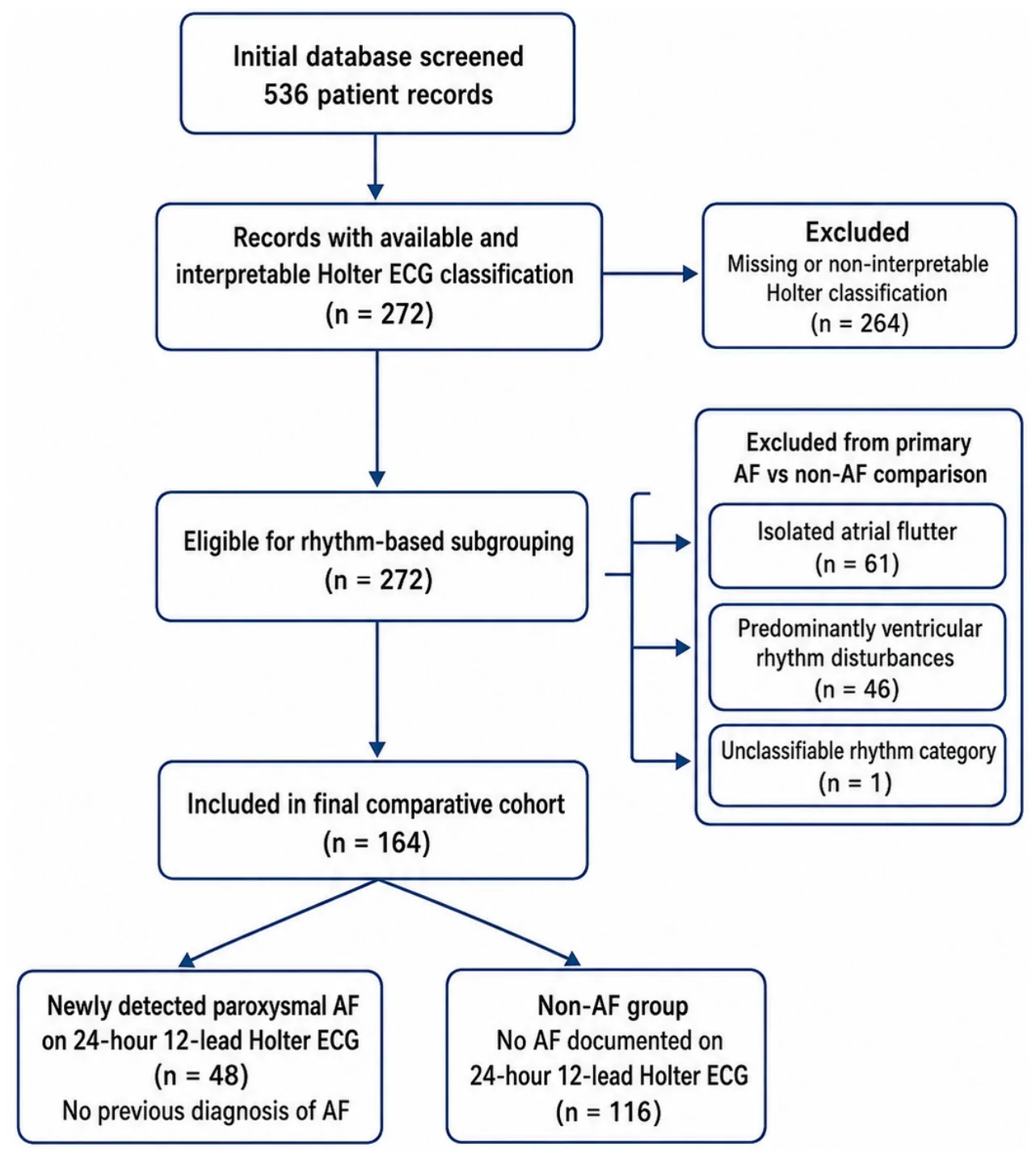
Flow-Chart Of Patient Selection. All included patients were referred to 24 h ECG-Holter due to palpitations and/or chest pain. No ischemic ECG changes were identified during the evaluation. AF = atrial fibrillation.

Patient selection process is better visualised in Figure 1.

Table 1 summarizes the baseline structure of the cohort and the availability of the main study domains. Patients with AF were significantly older than those without AF, while sex distribution did not differ significantly between groups. Data completeness was uneven across domains. Complete spirometric data were available in 24 AF and 42 non-AF patients, NT-proBNP values in 20 AF and 40 non-AF patients, and a core set of left atrial echocardiographic variables in 28 AF and 52 non-AF patients. Only 24 patients overall had simultaneous data from all three domains, which limited any fully integrated three-domain modeling to exploratory analyses only.

Before the strict spreadsheet-based comparison, the separate spirometry summary extracted from the same project showed negative correlations between AFib and the main respiratory parameters, namely FVC (r = −0.194), FEV1 (r = −0.183), and PEF (r = −0.112), with a lower mean FVC in AFib subjects than in non-AFib subjects (99% vs. 106%).

### 3.2. Spirometric and Biochemical Findings

The comparative analysis of the strict AF versus non-AF subset is shown in Table 2. In this complete-case subset, spirometric parameters were numerically close between groups and did not reach statistical significance. Median FVC was 104.5% in the AF group versus 102.0% in the non-AF group (*p* = 0.680). Median FEV1 was 102.0% versus 98.0% (*p* > 0.05), while median PEF was 96.0% versus 93.0% (*p* > 0.05).

Among the biochemical variables, the most robust signal was observed for NT-proBNP, which was markedly higher in the AF group. Median NT-proBNP was 684.5 pg/mL in AF patients compared with 62.3 pg/mL in non-AF patients (*p* = 0.001). Inflammatory markers showed a less pronounced separation between groups. Median hs-CRP was higher in AF patients, but without statistical significance (0.99 vs. 0.50 mg/L, *p* = 0.479). Similarly, the neutrophil/lymphocyte ratio was slightly higher in AF (2.08 vs. 1.89, *p* = 0.179). Homocysteine values were also higher in AF patients, but showed a nonsignificant trend (18.40 vs. 10.55 µmol/L, *p* = 0.062).

To explore whether the respiratory signal was diluted by exclusion of flutter cases, a supplementary grouping including any atrial arrhythmia (AF and/or flutter) was also reviewed. In that broader atrial arrhythmia subgroup, spirometric values were directionally lower than in the non-arrhythmic group, with median FVC 96.5% vs. 102.0%, median FEV1 94.0% vs. 98.0%, and median PEF 89.0% vs. 93.0%, although these comparisons also remained non-significant.

Because NT-proBNP may be influenced by several non-arrhythmic factors, an adjusted analysis was performed. In the multivariable logistic regression model, log-transformed NT-proBNP remained significantly associated with newly detected paroxysmal AF after adjustment for age, renal dysfunction, and chronic pulmonary disease. The adjusted association remained statistically significant [aOR = 3.12, 95% CI 1.42–6.86, *p* = 0.005], indicating that the NT-proBNP signal was not explained solely by these potential confounders.

### 3.3. Echocardiographic Findings

The echocardiographic comparison is presented in Table 3. In the available left atrial dataset, no structural or functional echocardiographic variable reached statistical significance in the AF versus non-AF comparison. Median anteroposterior left atrial diameter was 3.60 cm in AF patients and 3.47 cm in non-AF patients (*p* = 0.605). Median left atrial area at end-diastole was 15.38 cm^2^ in AF and 15.57 cm^2^ in non-AF (*p* = 0.477). Median left atrial volume at end-diastole was 40.56 mL versus 39.85 mL (*p* = 0.947), while median left atrial volume at end-systole was 56.95 mL versus 55.40 mL (*p* = 0.487).

Functional parameters showed the same pattern. Median left atrial ejection fraction was 44.55% in AF and 30.16% in non-AF patients, but the difference did not reach statistical significance (*p* = 0.169). Likewise, medial e’ values were nearly identical between groups (5.56 vs. 5.04 cm/s, *p* = 0.992). Taken together, these findings suggest that, within the smaller echocardiographic subgroup, the structural atrial signal was weaker than the biochemical NT-proBNP signal.

Transmitral A-wave and medial a′ findings are summarized in Table 4. Their higher rate of absence/non-measurability and lower measurable values in the AF group suggest impaired atrial mechanical contraction in patients with newly detected paroxysmal AF.

### 3.4. Exploratory Cross-Domain Correlations

Spirometry parameters showed inverse associations with NT-proBNP, with the strongest relationship observed for FEV1 (rho = −0.436, n = 32, *p* = 0.013). FVC also showed an inverse but non-significant association with NT-proBNP (rho = −0.323, n = 32, *p* = 0.071), while PEF was not significantly correlated with NT-proBNP (rho = −0.214, n = 32, *p* = 0.240). In relation to left atrial ejection fraction, FVC, FEV1, and PEF showed positive correlations, with rho values of 0.482, 0.568, and 0.345, respectively. These correlations reached statistical significance for FVC (*p* = 0.004), FEV1 (*p* < 0.001), and PEF (*p* = 0.046), as shown in Table 5.

**Figure 2:**
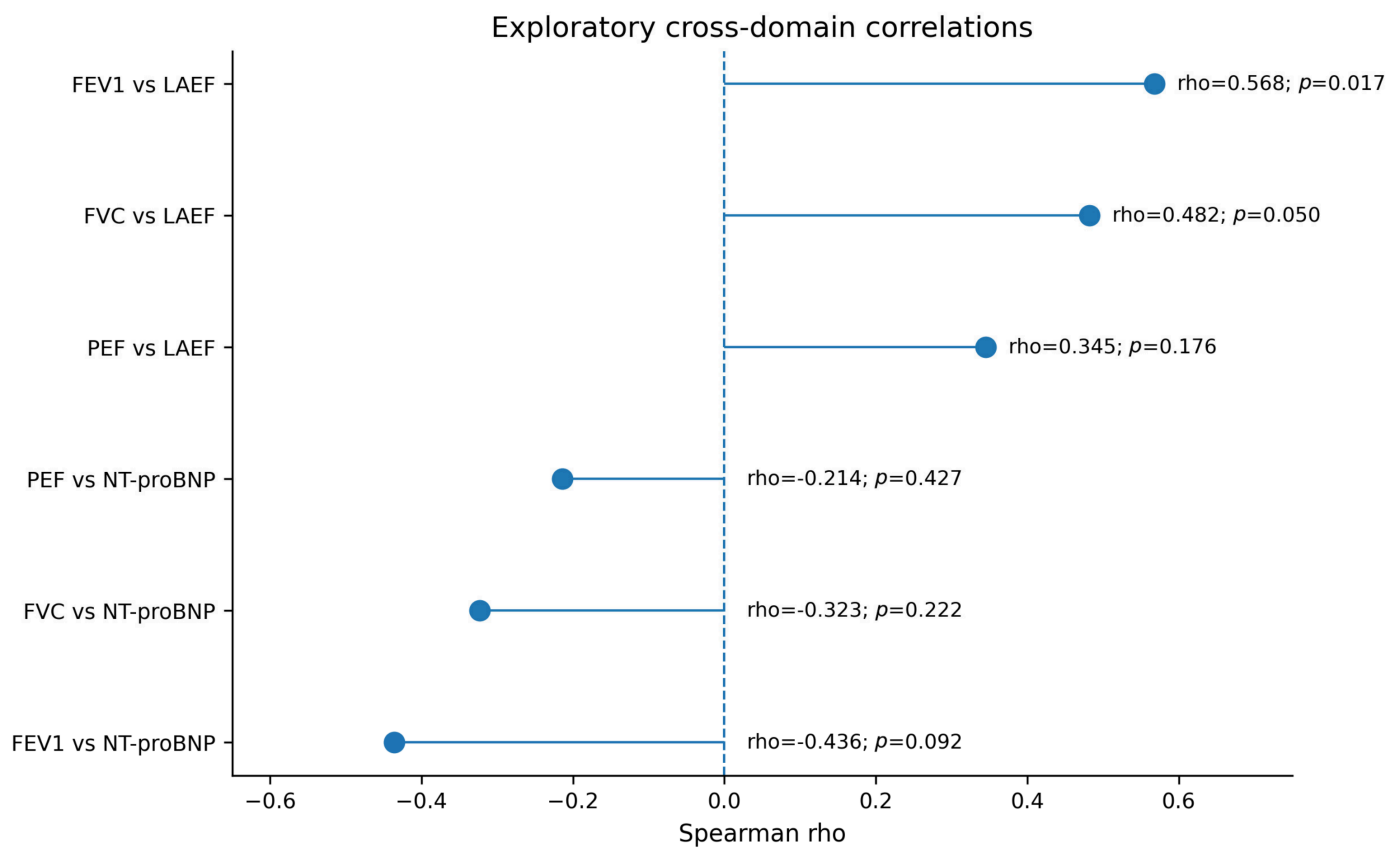
Lollipop plot showing Spearman correlation coefficients for the exploratory associations between spirometric variables, NT-proBNP, and left atrial ejection fraction in complete-case subsets. Positive coefficients indicate direct associations, while negative coefficients indicate inverse associations.

These exploratory findings suggest that the respiratory component may still carry physiologic relevance in the integrated cardiopulmonary phenotype, even though the between-group spirometric comparisons were attenuated in the stricter AF-only complete-case analysis. This is better visualised in Figure 2.

## 4. Discussion

### 4.1. Interpretation of the Main Findings

The main finding of the present study is that NT-proBNP was the clearest marker associated with atrial fibrillation in our cohort, whereas the spirometric and conventional echocardiographic signals were more modest. This hierarchy is consistent with the prior AF biomarker literature. In a large derivation and replication analysis, B-type natriuretic peptide improved AF risk prediction beyond clinical variables, whereas CRP added little incremental value, supporting the idea that natriuretic peptides are much closer to the core atrial/hemodynamic substrate than inflammatory markers alone [19]. A later multimarker study reached a very similar conclusion, showing that NT-proBNP was the strongest predictor of incident AF across two cohorts and improved discrimination when added to standard risk factors [4]. More recently, screening-oriented data have also shown that the prevalence of screening-detected AF is very low in participants with low NT-proBNP, and substantially higher when NT-proBNP is elevated, reinforcing its value as a clinically useful enrichment marker [5].

Importantly, this NT-proBNP signal persisted in the adjusted analysis. After accounting for age, renal dysfunction, and chronic pulmonary disease, NT-proBNP remained significantly associated with newly detected paroxysmal AF. This is clinically relevant because these variables are common determinants of NT-proBNP elevation [3,4,20], and could otherwise have explained part of the observed difference between groups.

The respiratory results should be interpreted differently. In our dataset, the spirometric signal was present, but weaker than the NT-proBNP signal. That does not make it biologically irrelevant. Earlier population work from the Copenhagen City Heart Study showed that reduced FEV1 was associated with a higher risk of subsequent AF hospitalization, even after adjustment for major cardiovascular covariates [21]. The Malmö Preventive Project later confirmed that impaired lung function, including lower FEV1 and FVC, independently predicted incident AF over long follow-up [22]. More recent cohort data have again linked reduced ventilatory parameters to incident AF independently of age, sex, smoking, and conventional cardiovascular risk factors [23]. Taken together, this makes the weaker respiratory signal in our strict complete-case analysis plausible rather than contradictory: lung function appears to be an associated layer of the AF phenotype, but not necessarily the dominant one in a smaller retrospective cohort.

The echocardiographic findings should also be read in context. Conventional left atrial dimensions are useful, but they do not fully describe atrial disease. The recent atrial cardiomyopathy literature emphasizes that left atrial dysfunction may precede overt enlargement, so a dataset built mainly on standard size and volume measurements can easily underestimate the true atrial substrate [24,25]. This is supported by population imaging data showing that left atrial strain predicts incident AF in the general population, suggesting that functional atrial impairment may be more sensitive than chamber dimensions alone [1,26,27].

Overall, our results support a clinically coherent interpretation. In this cohort, the biochemical domain carried the strongest AF signal, the respiratory domain remained suggestive and directionally consistent with the prior spirometric literature, and the echocardiographic domain was probably limited by the use of conventional atrial metrics rather than by the absence of atrial remodeling itself [19,22].

### 4.2. The Respiratory Component of the AF Phenotype

The respiratory findings in our cohort are better interpreted as a supportive phenotypic signal than as a primary discriminator of AF. This is still consistent with the literature. In the Malmö Preventive Project, lower baseline FEV1 and FVC were independently associated with incident AF over long follow-up, even after adjustment for smoking and other cardiovascular risk factors [22]. Earlier population data also linked reduced lung function to later AF hospitalization, supporting a stable association between impaired ventilatory performance and AF risk.

Mechanistically, this link is plausible. Reduced spirometric values may reflect not only concomitant lung disease, but also a broader milieu of hypoxia, inflammation, autonomic imbalance, and altered cardiopulmonary loading, all of which can favor atrial remodeling and arrhythmogenesis. Reviews focused on COPD and AF have similarly emphasized that pulmonary disease is not just a comorbidity, but part of a clinically relevant AF substrate that may affect progression and treatment response [10,28].

The practical implication is modest but important. In our study, spirometry was not strong enough to function as a stand-alone AF marker. Still, it may add useful functional characterization, especially in patients with suspected cardiopulmonary overlap. This fits recent Europace data showing that structured airflow screening can be successfully integrated into AF pathways and may uncover clinically relevant respiratory impairment that would otherwise remain unrecognized [29].

### 4.3. Biomarker Hierarchy and the Central Role of NT-proBNP

Within the biomarker panel, NT-proBNP was clearly the strongest signal in our cohort. This matches the prior AF literature. In a multimarker analysis, NT-proBNP was the strongest predictor of incident AF across two cohorts and improved prediction beyond standard clinical variables [4]. In community-based risk modeling, BNP improved AF risk stratification more clearly than CRP, again suggesting that natriuretic peptides are closer to the core atrial-hemodynamic substrate than inflammatory markers alone [19].

NT-proBNP remained significantly associated with newly detected AF after adjustment for age, renal dysfunction, and chronic pulmonary disease, suggesting that its elevation was not explained solely by these comorbidities. Heart failure status was not available for all patients and could not be entered as a separate covariate; given its strong mechanistic link to NT-proBNP, this is acknowledged as a limitation of the adjusted model.

Our weaker results for hs-CRP, N/L ratio, and homocysteine are therefore not surprising. Inflammatory markers are biologically relevant in AF, but their association is usually less robust and less consistent than that of natriuretic peptides. Recent inflammatory proteomics work still confirmed NT-proBNP as a strong AF predictor, even when broader inflammatory signatures were evaluated [30].

Clinically, this makes NT-proBNP the most credible biochemical anchor of the present article. It likely reflects atrial stretch, pressure load, and underlying atrial disease more directly than the other biomarkers we tested. This also keeps the internal logic of the paper clean: the integrated profile is centered on hemodynamic stress, while inflammation remains a secondary, supportive layer rather than the main biochemical driver [5,31].

### 4.4. The Echocardiographic Signal and the Limits of Conventional Atrial Assessment

The weaker echocardiographic signal in our cohort should be interpreted cautiously. In AF, atrial disease does not begin only when the left atrium becomes visibly enlarged. Studies on atrial mechanics show that left atrial dysfunction may precede left atrial enlargement, which means that standard diameter or volume measurements can miss earlier remodeling stages [24,32]. Notably, atrial functional impairment in AF is not confined to the left chamber: right atrial strain has also been proposed as a sensitive marker of atrial myopathy that complements left atrial assessment, and future iterations of this model should consider a biatrial functional approach.

This is why the recent literature has moved from simple atrial size toward atrial functional assessment, especially left atrial strain. In the Copenhagen City Heart Study, reduced left atrial strain predicted incident AF in the general population [1]. In older adults, lower left atrial strain and strain rate were also reported to be stronger predictors of AF than morphologic parameters such as left atrial volume [6].

For our study, the implication is mainly methodological. The absence of a strong signal from conventional atrial measurements does not argue against atrial remodeling; rather, it suggests that the available echo variables were probably less sensitive than NT-proBNP for capturing the relevant substrate in this retrospective cohort. This interpretation fits the current concept of atrial cardiomyopathy, in which functional impairment may be present before clear structural enlargement becomes evident.

The analysis of A wave and medial a′ provides an important physiological clarification. Both parameters reflect the active atrial contribution to ventricular filling. Therefore, they may be absent when the patient is in AF during echocardiographic acquisition and may be reduced in patients with impaired atrial mechanical function. In our cohort, A wave and medial a′ were more frequently absent or not measurable in patients with newly detected paroxysmal AF, and measurable values were lower than in the non-AF group.

### 4.5. Clinical Implications of the Integrated Cardiopulmonary Model

The main practical implication of our findings is that NT-proBNP appears to be the most useful first-line signal within this multimodal profile. In AF screening research, natriuretic peptide enrichment has already been used to identify patients with a higher diagnostic yield on subsequent rhythm monitoring, as shown in STROKESTOP II [33]. In the present cohort, this supports a simple clinical logic: when AF is suspected, NT-proBNP may help identify patients with a stronger atrial stress phenotype, while the other domains can refine characterization rather than replace rhythm documentation.

The spirometric layer is better interpreted as complementary. Our respiratory results do not support spirometry as a stand-alone AF marker, but they do suggest that respiratory assessment may add useful functional information in patients with suspected cardiopulmonary overlap. This is clinically relevant because structured airflow screening has already been successfully embedded into AF outpatient care and can uncover otherwise unrecognized obstructive disease [29].

**Figure 3:**
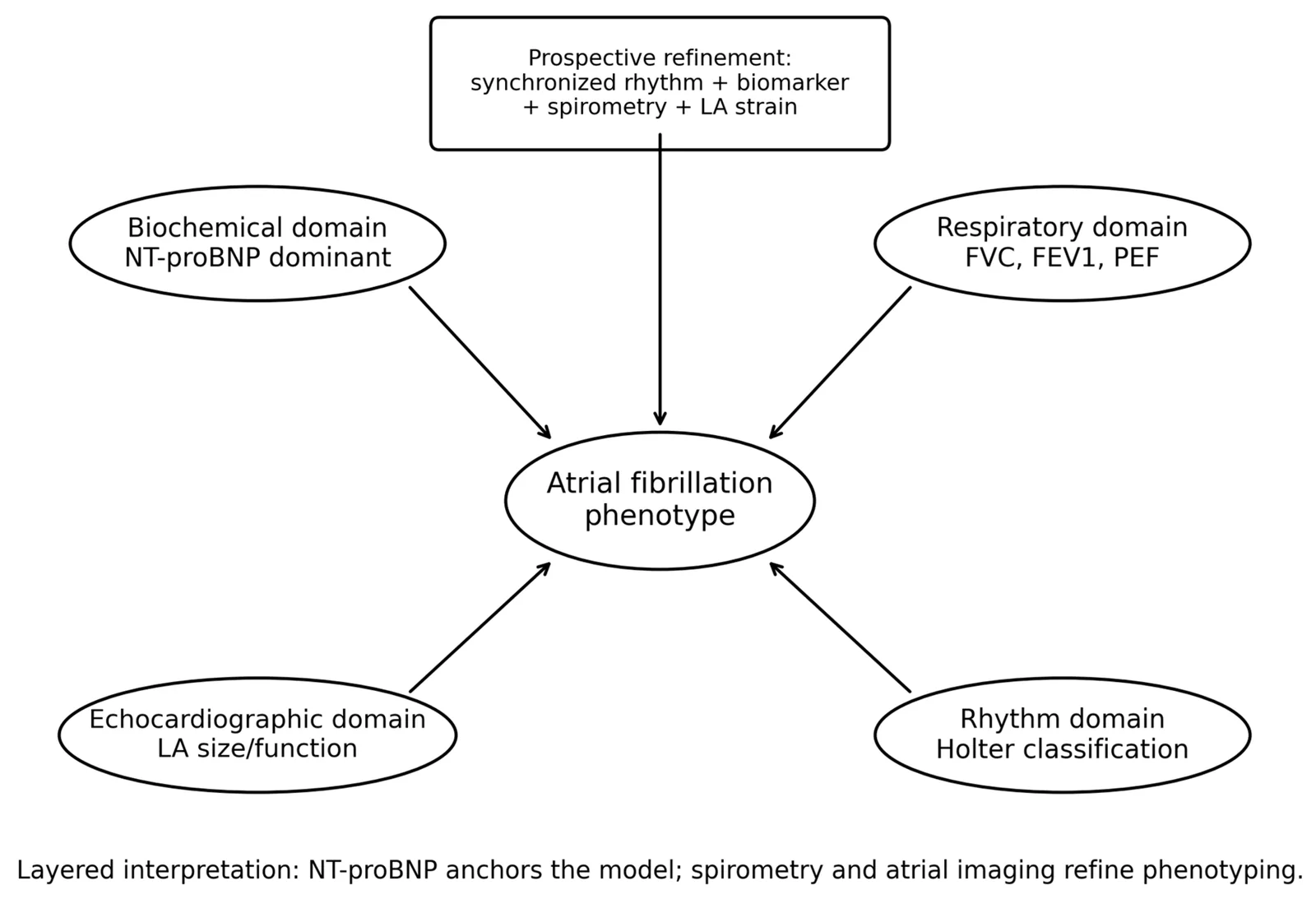
Conceptual model summarizing the proposed layered interpretation of AF as a cardiopulmonary phenotype integrating biochemical stress, spirometric performance, atrial structural/functional assessment, and rhythm documentation.

Overall, our data favor a layered rather than a single-test approach (Figure 3). This fits the broader move toward integrated AF care, where comorbidity assessment is part of routine management rather than an afterthought. Observational and trial data on integrated AF pathways have shown better outcomes when AF is managed together with its comorbidity burden and overall clinical profile [34,35]. Similar layered decision-making algorithms have been proposed in other clinical settings, where risk is not defined by a single measurement but by the interaction between multiple factors [36,37].

The most clinically relevant implication is not that NT-proBNP or spirometry can diagnose AF, but that they may help refine the evaluation of patients with suspected paroxysmal AF. In symptomatic patients with palpitations and/or chest pain, especially when traditional AF risk factors are also present, the association between elevated NT-proBNP and concordant spirometric impairment may act as an additive enrichment signal. Such a profile may justify repeated or prolonged rhythm monitoring beyond the initial 24 h This framing is consistent with the broader first-diagnosed AF literature, which describes a heterogeneous clinical profile and outcomes at initial diagnosis and emphasizes early, integrated management. Holter ECG, because short monitoring may miss intermittent paroxysmal AF. Therefore, the proposed cardiopulmonary profile should be viewed as a complementary tool for deciding who requires longer surveillance, not as a replacement for ECG documentation.

### 4.6. Study Limitations

This study has several limitations. First, it was a retrospective, single-center analysis, so it cannot support causal inference and is vulnerable to selection bias. Second, although the original worksheet was larger, the final AF vs. non-AF cohort became much smaller after rhythm reclassification and exclusion of incomplete records. This was even more evident in the complete-case analyses, where the overlap between spirometry, NT-proBNP, and echocardiography was limited. For this reason, the present results should be interpreted as hypothesis-generating rather than definitive.

AF was identified using 24 h Holter ECG monitoring; therefore, patients classified as non-AF may still have had very low-burden or intermittent AF not captured during the recording window. Although all AF cases were newly detected and paroxysmal, the retrospective design does not allow assessment of AF burden over time.

A second limitation is the depth of phenotyping, especially in the echocardiographic domain. Our analysis relied mainly on conventional left atrial measurements, while more sensitive functional metrics were not systematically available. This is relevant because left atrial strain predicts incident AF and may detect atrial dysfunction earlier than simple chamber size or volume alone [1,38].

A third limitation concerns AF ascertainment. Classification was based on the available Holter data, but shorter monitoring strategies can miss intermittent or low-burden AF. Europace reviews have emphasized that longer or repeated monitoring increases arrhythmia detection compared with standard short-duration Holter recordings [39,40].

Finally, the spirometric subgroup was relatively small, which likely reduced statistical power. This probably explains why the respiratory signal remained directionally consistent, but not uniformly significant, in the stricter complete-case comparison. Several additional limitations should be acknowledged. The overall cohort available for complete-case analyses was small relative to the original 536 screened records, and only 24 patients had simultaneous spirometry, NT-proBNP, and echocardiographic data; the pattern of missing data across domains was not formally characterized, patients with complete versus incomplete data were not systematically compared, and no sensitivity analysis for missing data was performed, so selection bias cannot be excluded and the findings of the fully integrated three-domain analysis in particular should be read as preliminary.

Baseline characterization was also incomplete: smoking status, body mass index, height, a formal spirometric definition of chronic pulmonary disease, heart failure subtype (HFpEF/HFmrEF/HFrEF), COPD prevalence, concomitant medication, and the exact timing of NT-proBNP sampling relative to the index Holter recording were not consistently available in the source database, which limits the interpretation of both the descriptive comparison and the adjusted NT-proBNP model.

Spiroergometry, which may be more sensitive than resting spirometry for detecting a respiratory contribution to AF risk, was not performed in this cohort and is proposed as a direction for future work. Finally, the “additive” value of concordant spirometric impairment and NT-proBNP elevation was not tested by comparing clinical models with and without these variables, and should be regarded as hypothesis-generating rather than demonstrated.

### 4.7. Future Directions

The next step should be a prospective study with synchronized data collection, so that Holter monitoring, spirometry, NT-proBNP, and echocardiography are performed in the same clinical window. This would reduce the missing-data overlap that limited the present analysis and would allow the integrated cardiopulmonary hypothesis to be tested more rigorously. Because short monitoring can miss intermittent AF, future protocols should also consider longer rhythm monitoring, which has a higher detection yield than conventional short-duration Holter strategies [40,41].

A second direction is to improve the echocardiographic sensitivity of the model. Conventional left atrial size is useful, but newer studies show that left atrial strain predicts incident AF and may outperform morphologic parameters for earlier substrate detection. Future versions of this project should therefore include at least one atrial functional metric, not only chamber dimensions or volumes [1,42,43].

Finally, a stronger future model should remain focused rather than oversized. In our dataset, NT-proBNP was the most robust biochemical signal, and NT-proBNP-enriched strategies have already shown value in AF detection pathways. A practical follow-up design would combine one biomarker, one atrial functional echo parameter, and one spirometric variable. This would fit current moves toward integrated AF care and phenotyping, while keeping the model clinically usable [33,44,45].

This pragmatic, focused approach is consistent with the broader move toward parsimonious, clinically interpretable risk-stratification models built from routinely available clinical variables.

## 5. Conclusions

In this retrospective cohort of patients referred for 24 h 12-lead Holter ECG monitoring because of palpitations and/or chest pain, newly detected paroxysmal AF was most strongly associated with NT-proBNP. Spirometric findings were weaker, but remained directionally consistent with a broader cardiopulmonary phenotype. Conventional echocardiographic parameters were less discriminative, probably because standard atrial measurements are less sensitive than functional atrial markers.

These findings should not be interpreted as supporting NT-proBNP or spirometry as stand-alone diagnostic tests for AF. Instead, they suggest that elevated NT-proBNP, especially when accompanied by concordant spirometric impairment, may provide additive information in symptomatic patients with suspected paroxysmal AF. Alongside traditional risk factors, this profile may help identify patients who could benefit from repeated or prolonged rhythm monitoring beyond 24 h.

## Figures and Tables

**Table 1:** Baseline characteristics and data availability in the AF versus non-AF cohort.

Variable	AF	non-AF
Number of patients, n	48	116
Age, years	69.50 (63.03–74.41)	59.32 (53.81–61.27)
Male sex, n (%)	12 (25.0)	44 (37.9)
Complete spirometry available, n (%)	24 (50.0)	42 (36.2)
NT-proBNP available, n (%)	20 (41.7)	40 (34.5)
Core left atrial echo availble, n (%)	28 (58.3)	52 (44.8)

Data are shown as median (IQR) unless otherwise specified. Core left atrial echo availability was defined as simultaneous availability of ASps, left atrial area at end-diastole, left atrial volume at end-diastole, and left atrial ejection fraction.

**Table 2:** Spirometric and biochemical variables in patients with and without AF.

Variable	AF	non-AF	*p*
FVC, % predicted	104.50 (92.00–115.25)	102.00 (94.00–119.00)	>0.05
FEV1, % predicted	102.00 (91.75–112.25)	98.00 (92.00–107.00)	>0.05
PEF, % predicted	96.00 (67.00–106.50)	93.00 (72.00–108.00)	>0.05
NT-proBNP, pg/mL	684.50 (255.75–4806.50)	62.25 (48.00–132.00)	0.001 *
hs-CRP, mg/L	0.99 (0.29–4.22)	0.50 (0.26–2.60)	>0.05
Neutrophil/lymphocyte ratio	2.08 (1.81–2.64)	1.89 (1.48–2.31)	>0.05
Homocysteine, µmol/L	18.40 (14.24–19.93)	10.55 (9.16–13.65)	>0.05

Values are presented as median (IQR). *p* values were calculated with the Mann–Whitney U test. * *p* < 0.05.

**Table 3:** Echocardiographic variables in patients with and without AF.

Variable	AF	non-AF	*p*
Left atrial AP diameter, cm	3.60 (3.31–3.89)	3.47 (3.01–4.22)	0.605
Left atrial area at end-diastole, cm^2^	15.38 (13.85–19.21)	15.57 (12.11–17.23)	0.477
Left atrial volume at end-diastole, mL	40.56 (29.39–51.12)	39.85 (30.77–49.87)	0.947
Left atrial volume at end-systole, mL	56.95 (46.68–83.78)	55.40 (41.99–73.86)	0.487
Left atrial ejection fraction, %	44.55 (35.70–52.56)	30.16 (19.36–46.27)	0.169
Medial e’, cm/s	5.56 (0.00–8.03)	5.04 (0.00–8.21)	0.992

Values are presented as median (IQR). *p* values were calculated with the Mann–Whitney U test. Diastole and systole refer to the ventricular cycle. Left atrial minimum volume, measured at end-ventricular diastole, mL. Left atrial maximum volume, measured at end-ventricular systole, mL.

**Table 4:** Transmitral A-wave and medial a′ findings.

Variable	AF	non-AF	*p*
Transmitral A wave absent/not measurable, n (%)	12/28 (42.9)	0/52 (0.0)	<0.001
Transmitral A-wave velocity, cm/s	54.0 (43.0–68.0)	78.0 (64.0–92.0)	0.006
Medial a′ absent/not measurable, n (%)	11/28 (39.3)	0/52 (0.0)	<0.001
Medial a′ velocity, cm/s	5.2 (4.1–6.6)	7.4 (5.8–8.9)	0.021

Values are presented as median (IQR) unless otherwise specified. A-wave and a′ velocities were analyzed only in patients in whom atrial contraction waves were measurable. Absence or non-measurability of A wave/a′ was reported separately because these parameters may be absent when the patient is in AF during echocardiographic acquisition.

**Table 5:** Exploratory cross-domain correlations in complete-case subsets.

Pair	n	Spearman rho	*p*
FVC vs. NT-proBNP	32	−0.323	0.071
FEV1 vs. NT-proBNP	32	−0.436	0.013 *
PEF vs. NT-proBNP	32	−0.214	0.240
FVC vs. left atrial ejection fraction	34	0.482	0.004 *
FEV1 vs. left atrial ejection fraction	34	0.568	<0.001 *
PEF vs. left atrial ejection fraction	34	0.345	0.046 *

Correlations were calculated using Spearman rank analysis in patients with simultaneous availability of both variables. * = statistical significance.

## Data Availability

The original contributions presented in this study are included in the article. Further inquiries can be directed to the corresponding author.

## Abbreviations

The following abbreviations are used in this manuscript:

AF	Atrial fibrillation
AFib	Atrial fibrillation
AP	Anteroposterior
ATS	American Thoracic Society
BNP	B-type natriuretic peptide
COPD	Chronic obstructive pulmonary disease
ECG	Electrocardiogram
ERS	European Respiratory Society
ESC	European Society of Cardiology
FEV1	Forced expiratory volume in 1 s
FVC	Forced vital capacity
GDPR	General Data Protection Regulation
hs-CRP	High-sensitivity C-reactive protein
IQR	Interquartile range
LA	Left atrial
N/L ratio	Neutrophil-to-lymphocyte ratio
NT-proBNP	N-terminal pro-B-type natriuretic peptide
PEF	Peak expiratory flow
SD	Standard deviation
